# A heart-stopping procedure: severe esophageal injury arising from difficulty in withdrawal of a motorized spiral enteroscope

**DOI:** 10.1055/a-2320-4024

**Published:** 2024-06-05

**Authors:** Nicolás Felipe Prado Troya, María Luisa López García, Leire Irusta Olano, Ángel José Calderón García, Ana Belen Díaz-Roca, Ainara Merino Zubizarreta, Pilar Cabezudo Gil

**Affiliations:** 1 16831 Aparato Digestivo General, Basurto University Hospital, Bilbao, Spain

We present the case of an 83-year-old man, with a history of heart disease and duodenal ulcer, who was admitted for syncope and a 5-day history of melena and anemia.

After several radiologic and endoscopic explorations (gastroscopy, two capsule endoscopies, and a double-balloon enteroscopy), the second capsule endoscopy showed a submucosal lesion in the mid-ileum, so, as a last resort, the team decided to perform motorized spiral enteroscopy (MSE).

The enteroscopy was done under general anesthesia. A Dieulafoy lesion with active bleeding was observed in the mid-ileum, and was treated with argon plasma coagulation. A submucosal lesion that was not treated was also seen.

At the end of the procedure, we were confronted by an unexpected problem: the scope could not be withdrawn, neither forward nor backward movement being possible. After 20 minutes and following different interventions by different endoscopists, it was decided to hyperextend the patient’s neck and deflate the balloon from the orotracheal tube, permitting the appropriate withdrawal of the scope.

A gastroscope was used to inspect the resulting damage. Numerous hematomas and mucosal tearing throughout the esophagus could be seen, but no perforation was observed ( Video 1 ).

Second look at esophagus using a gastroscope, after challenging extraction of motorized spiral enteroscope in an 83-year-old man.Video 1

The patient was conscious when he left the room. After a 48-hour fast, an esophagogram was taken, and no perforation was seen. A progressive diet was started, the patient presented mild dysphagia, and was discharged from the hospital.

Reviewing the literature, no recent international papers have described complications similar to our case, though some have described mild esophageal lesions 1 .

In a meta-analysis published in September 2022 that included 9 studies with 959 patients, adverse events were seen in 17%, with only 1% considered to be serious (3 perforations, 2 pancreatitis, 6 hemorrhages) 2 . There were no deaths and, of course, no report of the complication described above.

In another systematic review and meta-analysis published in June 2023 that included 10 studies and 961 patients, adverse events were seen in 18.1%. These included 6 difficult withdrawals, all considered to be minor events, and 20 instances of esophageal abrasions (deep and superficial), also considered to be minor events 3 .

In July 2023, the manufacturers of the motorized spiral enteroscope recalled it from the market because of an unsuccessful withdrawal of the instrument that had required surgical intervention to remove it from the patient.

Endoscopy_UCTN_Code_TTT_1AP_2AD

## Notes

Endoscopy E-Videos https://eref.thieme.de/e-videos
*E-Videos* is an open access online section of the journal *Endoscopy* , reporting on interesting cases and new techniques in gastroenterological endoscopy. All papers include a high-quality video and are published with a Creative Commons CC-BY license. Endoscopy E-Videos qualify for HINARI discounts and waivers and eligibility is automatically checked during the submission process. We grant 100% waivers to articles whose corresponding authors are based in Group A countries and 50% waivers to those who are based in Group B countries as classified by Research4Life (see: https://www.research4life.org/access/eligibility/ ). This section has its own submission website at https://mc.manuscriptcentral.com/e-videos .
